# Risk Group Stratification for True Early Recurrence After Ablation for Hepatomas

**DOI:** 10.1155/cjgh/8778095

**Published:** 2026-06-28

**Authors:** Yi-Hao Yen, Kwong-Ming Kee, Chao-Hung Hung, Chien-Hung Chen, Tsung-Hui Hu, Jing-Houng Wang, Sheng-Nan Lu

**Affiliations:** ^1^ Division of Hepatogastroenterology, Department of Internal Medicine, Kaohsiung Chang Gung Memorial Hospital and, Chang Gung University College of Medicine, Kaohsiung, Taiwan, cgu.edu.tw

**Keywords:** early recurrence, hepatocellular carcinoma, overall survival, radiofrequency ablation

## Abstract

**Background:**

Hepatocellular carcinoma (HCC) is a lethal cancer. Early recurrence, i.e., recurrence within two years of curative treatment, is a major determinant of ultimate survival.

**Materials and Methods:**

We included 625 patients with newly diagnosed early‐stage HCC, i.e., Barcelona Clinic Liver Cancer (BCLC) Stage 0 or A, and Child–Pugh Class A liver disease who underwent percutaneous radiofrequency ablation (RFA) between 2011 and 2021 at our institution with a follow‐up period of > 2 years. The patients were divided into Group 1 (patients who developed nonlocal recurrence or died within two years after RFA; *n* = 300 [48.0%]) and Group 2 (patients who developed local recurrence within two years or were recurrence‐free and were alive for two years after RFA; *n* = 325 [52.0%]).

**Results:**

Multivariate analysis showed that a Model for End‐Stage Liver Disease (MELD) score of > 9, anti‐hepatitis C virus (HCV) positivity, the presence of image‐defined cirrhosis, treatment with antiviral therapies for hepatitis B virus or HCV, alpha‐fetoprotein ≥ 20 ng/mL, multiple tumors, and larger tumor size were independent factors associated with Group 1. A nomogram was developed based on these variables to predict Group 1, with a concordance index of 68.3% (95% CI = 64.1%–72.5%). The 10‐year overall survival of Group 1 was 28%, and that of Group 2 was 64%.

**Conclusion:**

We developed a nomogram to predict true early recurrence (i.e., nonlocal recurrence) of HCC after RFA.

## 1. Introduction

In Taiwan, hepatocellular carcinoma (HCC) is one of the leading causes of cancer‐related death [1]. Curative treatment options recommended for patients with very‐early‐/early‐stage HCC (Barcelona Clinic Liver Cancer [BCLC] 0/A) are liver resection (LR), liver transplantation, radiofrequency ablation (RFA), microwave ablation, and yttrium‐90 transarterial radioembolization [2]. Microwave ablation shows better local control of tumors sized 3–5 cm [3].

According to the European Association for the Study of the Liver (EASL) guideline recommendation, RFA is the standard of care for patients with BCLC 0 and A tumors not suitable for surgery [4]. However, the rate of recurrence of HCC after RFA is high [5]. HCC recurrence can be classified into early recurrence (< 2 years) and late recurrence (> 2 years) [6]. Early recurrence of HCC is generally considered a result of occult metastasis of the primary tumor, while late recurrence is considered a new carcinoma in the context of underlying liver disease [7]. This concept is only applicable to patients who undergo LR [8], which is oncologically advantageous for the complete resection of tumors. The local recurrence rate after LR is extremely low compared to that after RFA [9]. Local recurrence is due to incomplete ablation and thus cannot be regarded as true recurrence. HCC with early recurrence usually has a poor prognosis and, consequently, early recurrence is the main cause of survival of patients with HCC [10]. Therefore, we investigated predictors of patients with HCC who showed true early recurrence (i.e., nonlocal recurrence) after RFA.

## 2. Materials and Methods

**FIGURE 1:**
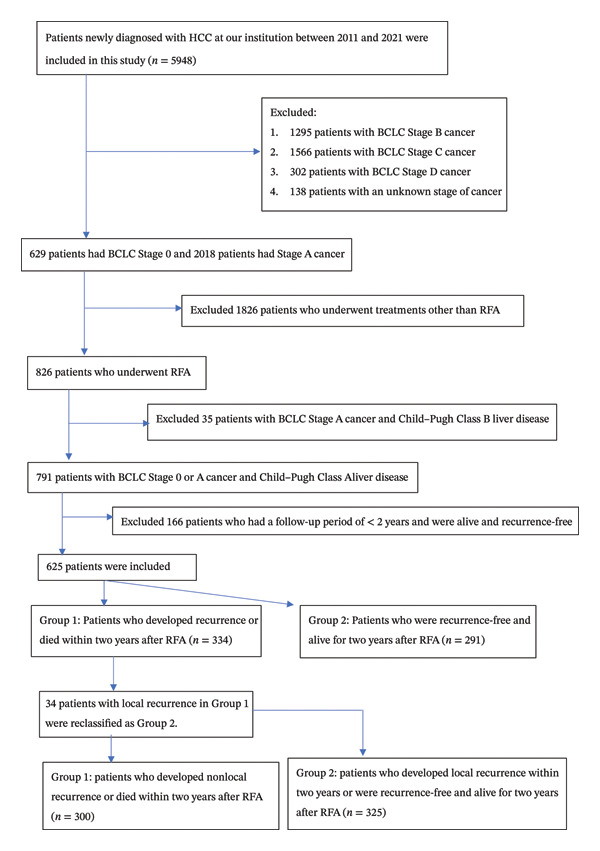
Flowchart of patient enrollment.

The Institutional Review Board of our institution approved this study (approved number: 202400371B0). The inclusion criterion was patients with BCLC Stage 0–A [11] HCC and Child–Pugh Class A liver disease [12] undergoing percutaneous RFA. The criterion for exclusion in this study was a follow‐up period of < 2 years and being alive and recurrence‐free. Patients who developed nonlocal recurrence or died within two years after RFA were classified as Group 1. Patients who developed local recurrence within two years or who were recurrence‐free and alive for two years after RFA were classified as Group 2. None of these patients received adjuvant therapy after RFA. The flowchart of patient enrollment is shown in Figure 1.

### 2.1. RFA Procedure

The RFA procedure was described in detail in our previous studies [13–15]. RFA was performed by multiple operators. The energy delivered during RFA and the duration of ablation were according to the manufacturers’ recommendations. For example, the most commonly used RFA needle in our institution was the cool‐tip needle (Cool‐tip RF Ablation System, Medtronic, Minneapolis, MN, USA). Percutaneous RFA was performed with a 17‐gauge cool‐tip needle with a two‐ or 3 cm exposed tip and a power generator. For the 2 cm exposed tip needle, an initial power output of 40 W was used, with the power increasing by 10 W per minute and a maximum power output of 200 W, and the ablation time was 6 min. For the 3‐cm exposed tip needle, the initial power output was 60 W, with the power increasing by 10 W per minute and a maximum power output of 200 W, and the ablation time was 12 min. RFA was performed under ultrasound guidance. The ablation zone was monitored using ultrasound. High echoic areas due to RFA‐induced microbubbles could help estimate the extent of ablation. The post‐procedure margin was visually confirmed.

### 2.2. Follow‐Up and Definition of Recurrence

Contrast‐enhanced computed tomography (CT) was performed to assess treatment effects one month after RFA. Ablation was considered to be complete if no enhanced area was demonstrated at the site of the index tumor. Incomplete ablation was retreated with RFA, ethanol injection, or transarterial chemoembolization. After complete ablation, all patients were followed up with testing of their serum AFP level plus ultrasound every three months. If recurrence was suspected from ultrasonography or AFP elevation during post‐RFA follow‐up, contrast‐enhanced CT was performed for a definite diagnosis. When ultrasonography or the AFP level did not indicate recurrence, whether to perform contrast‐enhanced CT depended on the clinical decision of the physician in charge. Some physicians used contrast‐enhanced CT as a surveillance tool for patients with a high risk of recurrence, for example, those with a large tumor, multiple tumors, or a high AFP level before RFA.

Recurrence of HCC after ablation was defined by image studies or pathology [4]. Recurrence was classified as local recurrence and intrahepatic distant recurrence (IDR). Local recurrence was defined as the presence of an enhancing tumor adjacent to the ablated area after achievement of a complete radiological response. IDR was defined as the presence of tumor recurrence in locations not adjacent to the ablated area [16]. In this study, 334 patients developed recurrence or died within two years after RFA, while the remaining 291 patients were recurrence‐free and alive for two years after RFA. Of the 334 patients who developed recurrence or died, 34 showed local recurrence within two years after RFA; the remaining 300 patients were designated as Group 1. The 34 patients with local recurrence within two years after RFA, plus the 291 patients who were recurrence‐free and alive for two years after RFA, were designated as Group 2 (*n* = 325).

Overall survival (OS) was defined as the time elapsed between the date of RFA and the date of the last medical visit or death. Patients who were lost to follow‐up were censored at their last medical visit.

### 2.3. Statistical Analyses

Differences in patient characteristics between groups were assessed using the Mann–Whitney *U* test for continuous variables and the chi‐square test for categorical variables. Survival rates were calculated using the Kaplan–Meier method and compared using the log‐rank test. The multivariable logistic regression applied a stepwise logistic model to analyze variables used in univariate analyses. A nomogram model was established based on the results of the multivariable logistic regression and validated using bootstrap resampling. Calibration was performed by plotting the predicted probability versus the observed probability of true early recurrence status. *p* values < 0.05 were considered statistically significant.

## 3. Results

### 3.1. Comparison of Patient Characteristics

We divided patients into Group 1, i.e., patients who developed nonlocal recurrence or died within two years after RFA (*n* = 300 [48.0%]), and Group 2, i.e., patients who developed local recurrence within two years after RFA and patients who were recurrence‐free and alive for two years after RFA (*n* = 325 [52.0%]). The proportion of patients with a Model for End‐Stage Liver Disease (MELD) score of > 9 was higher (*p* = 0.001), anti‐hepatitis C virus (HCV) positivity was higher (*p* = 0.009), cirrhosis was higher (*p* = 0.007), BCLC Stage A disease was higher (*p* = 0.006), AFP level of ≥ 20 ng/mL was higher (*p* = 0.002), and multiple tumors was higher (*p* = 0.016), and the proportion with hepatitis B surface antigen (HBsAg) positivity was lower (*p* = 0.002), and undergoing antiviral therapies for hepatitis B virus (HBV) (*p* = 0.02) and HCV (*p* = 0.045) was lower in Group 1 compared to Group 2. The median body mass index (BMI) was higher (0.015), and tumor size (*p* = 0.008) was larger in Group 1 compared to Group 2. The proportion of patients aged > 65 years, sex, and pathology diagnosis of HCC did not differ between the two groups (Table 1).

**TABLE 1:** Comparison of characteristics between patients who developed nonlocal recurrence or died within two years after radiofrequency ablation and patients who developed local recurrence within two years or were recurrence‐free and alive for two years after ablation.

	**Patients who developed nonlocal recurrence or died within 2 years after ablation, *n* = 300**	**Patients who developed local recurrence within 2 years or were recurrence-free and alive for 2 years after ablation, *n* = 325**	***p***

BMI (kg/m^2^)	25.5 (23.1–28.3)	24.9 (22.5–27.0)	0.015
MELD score > 9	144 (48.0%)	115 (35.4%)	0.001
Age > 65 years	145 (48.3%)	160 (49.2%)	0.823
Anti‐HCV positive	165 (55.0%)	145 (44.6%)	0.009
HBsAg positive	101 (33.7%)	148 (45.5%)	0.002
Presence of image‐defined cirrhosis	260 (86.7%)	255 (78.5%)	0.007
AFP (ng/mL)			0.002
< 20	169 (56.3%)	222 (68.3%)	
≧ 20	131 (43.7%)	103 (31.7%)	
Tumor number			0.016
Single	228 (76.0%)	272 (83.7%)	
Multiple	72 (24.0%)	53 (16.3%)	
Tumor size (cm)	2.4 (1.9–2.9)	2.1 (1.8–2.8)	0.008
Men	185 (61.7%)	206 (63.4%)	0.658
Received antiviral therapy for HBV	76 (25.3%)	120 (36.9%)	0.002
Received antiviral therapy for HCV	76 (25.3%)	106 (32.6%)	0.045
Method of HCC diagnosis			0.159
Image studies	140 (46.7%)	170 (52.3%)	
Pathology	160 (53.3%)	155 (47.7%)	
BCLC stage			0.006
0	92 (30.7%)	134 (41.2%)	
A	208 (69.3%)	191 (58.8%)	

*Note:* AFP, alpha‐fetoprotein; MELD, Model for End‐Stage Liver Disease; HBsAg, hepatitis B surface antigen; HCC, hepatocellular carcinoma.

Abbreviations: BCLC, Barcelona Clinic Liver Cancer; BMI, body mass index; HBV, hepatitis B virus; HCV, hepatitis C virus.

### 3.2. Variables Associated With Nonlocal Recurrence or Death Within Two Years After RFA

Univariate analysis showed that a MELD score of > 9 (odds ratio [OR] = 1.686; 95% confidence interval [CI] = 1.223–2.323; *p* = 0.001), anti‐HCV positivity (OR = 1.517; 95% CI = 1.107–2.080; *p* = 0.010), HBsAg positivity (OR = 0.607; 95% CI = 0.439–0.839; *p* = 0.003), image‐defined cirrhosis (OR = 1.784; 95% CI = 1.166–2.730; *p* = 0.008), AFP level of ≥ 20 ng/mL (OR = 1.671; 95% CI = 1.205–2.316; *p* = 0.002), tumor size increase per 1 cm (OR = 1.371; 95% CI = 1.102–1.706; *p* = 0.005), multiple tumors (OR = 1.621; 95% CI = 1.091–2.408; *p* = 0.017), treatment with antiviral therapies for HBV (OR = 0.580; 95% CI = 0.411–0.818; *p* = 0.002), and treatment with antiviral therapies for HCV (OR = 0.701; 95% CI = 0.495–0.993; *p* = 0.046) were associated with patients who showed nonlocal recurrence or died within two years after RFA. Multivariate analysis showed that a MELD score of > 9 (OR = 1.472; 95% CI = 1.050–2.065; *p* = 0.025), anti‐HCV positivity (OR = 2.035; 95% CI = 1.252–3.309; *p* = 0.004), image‐defined cirrhosis (OR = 1.704; 95% CI = 1.090–2.663; *p* = 0.019), AFP level of ≥ 20 ng/mL (OR = 1.537; 95% CI = 1.084–2.180; *p* = 0.016), multiple tumors (OR = 1.581; 95% CI = 1.043–2.395; *p* = 0.031), tumor size increase per 1 cm (OR = 1.333; 95% CI = 1.058–1.678; *p* = 0.015), treatment with antiviral therapies for HBV (OR = 0.609; 95% CI = 0.401–0.925; *p* = 0.020), and treatment with antiviral therapies for HCV (OR = 0.363; 95% CI = 0.223–0.592; *p* < 0.001) were associated with patients who developed nonlocal recurrence or died within two years after RFA (Table 2).

**TABLE 2:** Univariate and multivariate logistic regression analyses of factors associated with patients who developed nonlocal recurrence or died within two years after ablation.

Variables	Univariate		Multivariate	
	OR (95% CI)	*p*	OR (95% CI)	*p*
MELD score > vs. ≦ 9	1.686 (1.223–2.323)	0.001	1.472 (1.050–2.065)	0.025
Age > vs. ≦ 65 years	0.965 (0.705–1.321)	0.823		
Anti‐HCV positive vs. negative	1.517 (1.107–2.080)	0.010	2.035 (1.252–3.309)	0.004
HBsAg positive vs. negative	0.607 (0.439–0.839)	0.003		
Presence vs. absence of image‐defined cirrhosis	1.784 (1.166–2.730)	0.008	1.704 (1.090–2.663)	0.019
AFP ≧ vs. < 20 ng/mL	1.671 (1.205–2.316)	0.002	1.537 (1.084–2.180)	0.016
Tumor number multiple vs. single	1.621 (1.091–2.408)	0.017	1.581 (1.043–2.395)	0.031
Tumor size increase per 1 cm	1.371 (1.102–1.706)	0.005	1.333 (1.058–1.678)	0.015
Men vs. women	0.929 (0.672–1.285)	0.658		
Received antiviral therapy for HBV	0.580 (0.411–0.818)	0.002	0.609 (0.401–0.925)	0.020
Received antiviral therapy for HCV	0.701 (0.495–0.993)	0.046	0.363 (0.223–0.592)	< 0.001

*Note:* AFP, alpha‐fetoprotein; MELD, Model for End‐Stage Liver Disease; HBsAg, hepatitis B surface antigen; HCC, hepatocellular carcinoma.

Abbreviations: BCLC, Barcelona Clinic Liver Cancer; BMI, body mass index; HBV, hepatitis B virus; HCV, hepatitis C virus.

### 3.3. Establishment and Verification of the Nomogram

**FIGURE 2:**
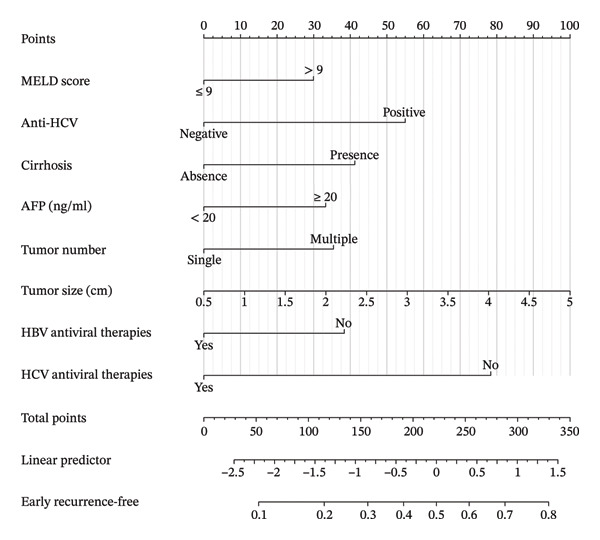
A nomogram to predict true early recurrence or death within two years after radiofrequency ablation.

**FIGURE 3:**
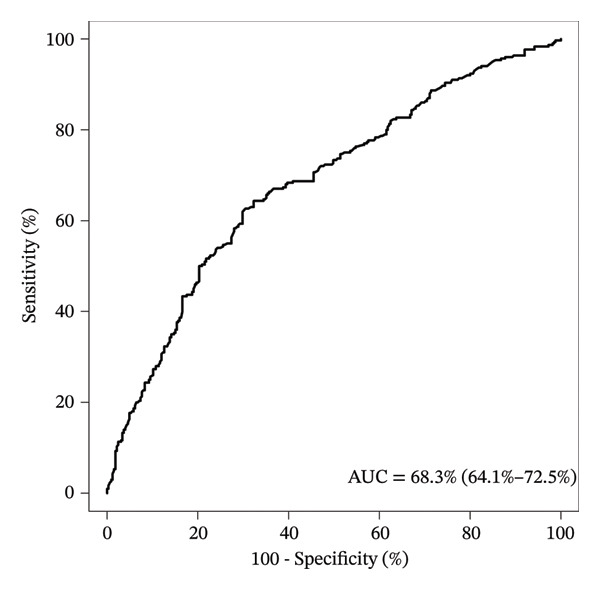
The area under the curve of the receiver operating characteristic curve of our nomogram.

**FIGURE 4:**
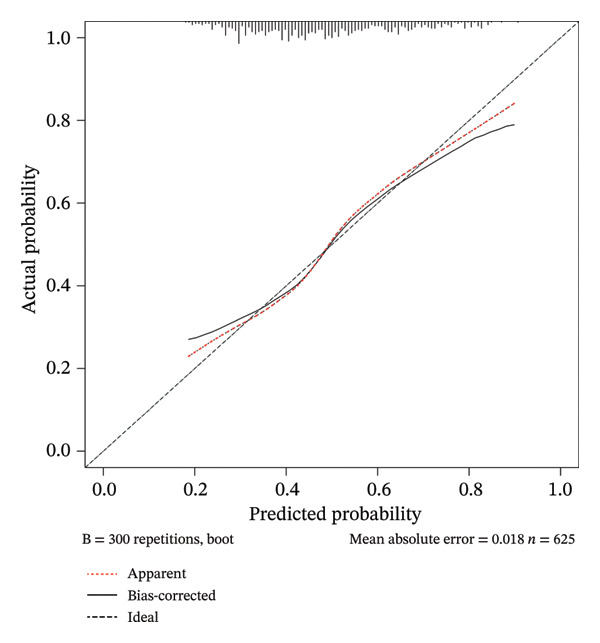
The model’s accuracy is visualized by comparing the predicted and the actual probabilities of true early recurrence or death within two years after radiofrequency ablation for hepatocellular carcinoma, showing the apparent predictive ability and bias due to overfitting. The relative prevalence of probability levels is indicated by the vertical lines at the top of the plot.

A nomogram including the MELD score, anti‐HCV positivity, image‐defined cirrhosis, AFP level, tumor size, tumor number, and treatment with antiviral therapies was constructed to predict nonlocal recurrence or death within two years after RFA (Figure 2). The C‐index of the nomogram was 68.3% (95% CI = 64.1%–72.5%) (Figure 3). The calibration plots showed overall high agreement between the predictions made by the model and observed outcomes (Figure 4).

### 3.4. Comparison of 10‐Year OS

**FIGURE 5:**
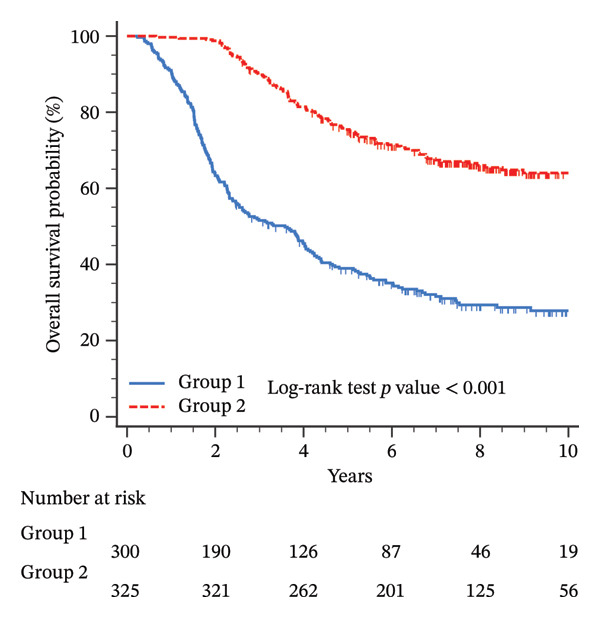
Ten‐year overall survival of patients with hepatocellular carcinoma who developed true recurrence or died within two years after radiofrequency ablation (RFA) (Group 1) and of patients who developed local recurrence within two years or who were recurrence‐free and alive for two years after RFA (Group 2).

The median (IQR) follow‐up period was 5.5 (2.5–8.3) years. The 10‐year OS of Group 1 patients was 28%, and that of Group 2 patients was 64% (*p* < 0.001) (Figure 5).

### 3.5. 10‐Year OS by Age at the Time of RFA

**FIGURE 6:**
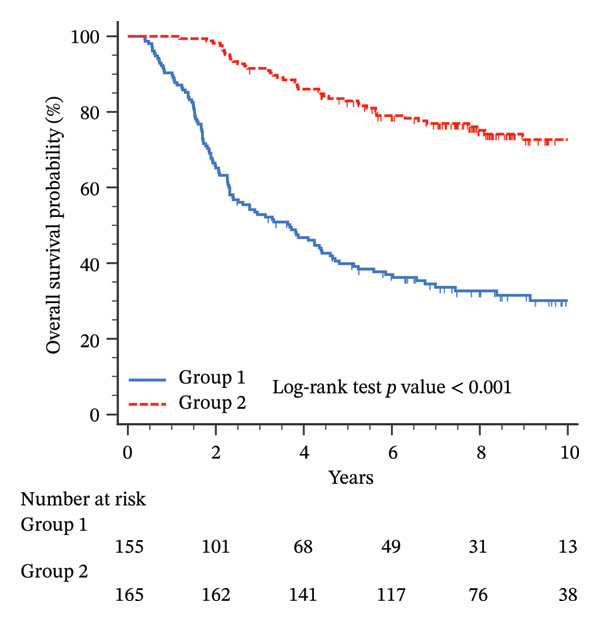
Ten‐year overall survival of patients with hepatocellular carcinoma aged ≤ 65 years at the time of radiofrequency ablation (RFA) who developed true recurrence or died within two years after RFA (Group 1) and of patients who developed local recurrence within two years or who were recurrence‐free and alive for two years after RFA (Group 2).

**FIGURE 7:**
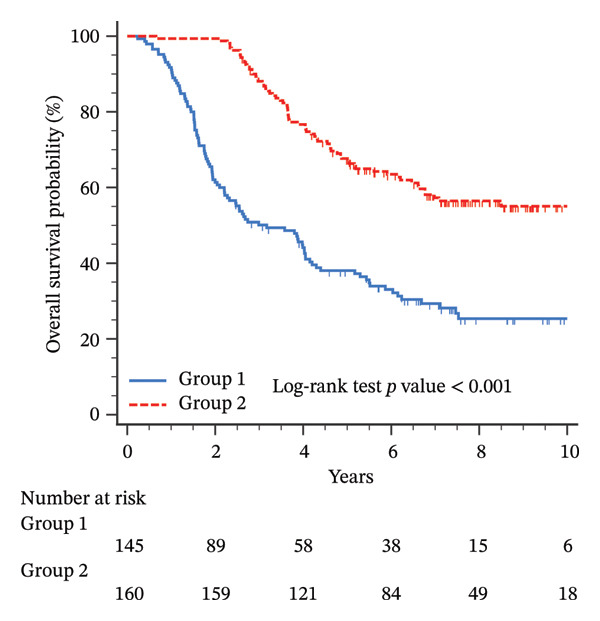
Ten‐year overall survival of patients with hepatocellular carcinoma aged > 65 years at the time of radiofrequency ablation (RFA) who developed true recurrence or died within two years after RFA (Group 1) and of patients who developed local recurrence within two years or who were recurrence‐free and alive for two years after RFA (Group 2).

Because life expectancy decreases as age increases, we divided patients into two groups based on their age at the time of RFA: > 65 years and ≤ 65 years. In the case of patients aged ≤ 65 years, the 10‐year OS of Group 1 patients was 30%, and that of Group 2 patients was 74% (*p* < 0.001) (Figure 6). In the case of patients aged > 65 years, the 10‐year OS of Group 1 patients was 24%, and that of Group 2 patients was 58% (*p* < 0.001) (Figure 7).

## 4. Discussion

Cancer patients frequently suffer from depressive disorders, which have a significant impact on their quality of life. Therefore, clinicians should communicate effectively and dynamically with cancer patients about prognosis [17]. In the present study, we developed a nomogram to predict true early recurrence after RFA based on the notion that early recurrence is the main cause of survival [10]. This nomogram could predict prognosis at baseline. Consequently, after two years of follow‐up, we were able to dynamically inform the patients about their prognosis according to their status of recurrence.

In the present study, 48% of the patients showed true early recurrence or died within two years after RFA. As expected, tumor‐related factors, including AFP level ≥ 20 ng/mL, multiple tumors, and larger tumor size, were associated with true early recurrence or death within two years after RFA.

Liver‐related factors, including a MELD score of > 9, presence of image‐defined cirrhosis, anti‐HCV positivity, and treatment with antiviral therapies, were also associated with true early recurrence or death within two years after RFA. A genome study reported that a significant fraction of early‐recurring tumors after LR are “de novo” tumors arising due to underlying liver disease [18], which may explain the results of our study. A previous study also showed that liver‐related factors, such as a higher albumin–bilirubin grade, are associated with early recurrence [7].

Li et al. reported that antiviral therapy was associated with reduced incidences of early recurrence after LR for HBV‐related HCC [19]. Multiple studies have shown that sustained virologic response (SVR) improved RFS in patients with HCV‐related HCC undergoing LR or RFA [20, 21]. Although these studies did not specifically analyze whether SVR improved early recurrence, the Kaplan–Meier plots indicated that SVR improved RFS during the first 2 years after LR or RFA [20, 21]. The results of our study are consistent with these previous findings [20, 21].

We included patients with Child–Pugh Class A liver disease in the present study. Traditionally, Child–Pugh Class A liver disease is subclassified as Child–Pugh score 5 or 6, with or without clinically significant portal hypertension and albumin–bilirubin Grade 1 or 2/3 [2]. Our HCC registry data lacked the abovementioned data. Therefore, we used a MELD score of > 9 or ≤ 9 to subclassify Child–Pugh Class A liver disease. This cutoff value was adopted from a previous study that enrolled 543 patients with chronic liver disease who underwent LR for HCC. A MELD score of > 9 is an independent factor associated with postoperative liver decompensation [22]. Therefore, we assumed that a MELD score of > 9 represented inadequate liver function reserve in patients with compensated liver disease. However, the MELD score is rarely used to predict the prognosis of patients with HCC undergoing RFA. Therefore, external validation of our results is needed.

The concept of our study is derived from conditional survival. Conditional survival, derived from the concept of conditional probability, is the probability that a patient who has survived for a specific period would still be alive during another fixed interval [23]. Previous studies used mortality rather than recurrence to predict conditional survival. In addition, these studies were mainly of patients with HCC undergoing LR [24, 25]. In contrast, we determined the probability that a patient who was truly recurrence‐free for two years after RFA would still be alive for another eight years.

The concept of our study was also derived from Cucchetti et al., who used data from 2523 patients undergoing LR for HCC to estimate the probability that LR would enable patients to achieve the same life expectancy as that of patients with chronic liver disease without HCC. The cure model suggests that LR can enable patients with HCC to achieve the same life expectancy as that of patients with chronic liver disease (i.e., which is defined as statistical cure) in 26.3% of cases. If a patient was alive without recurrence two years after surgery, his/her likelihood of being “cured” was about 50% [10]. In the present study, if a patient was alive without true recurrence two years after RFA, the 10‐year OS was 64% for the whole cohort. In the case of patients aged ≤ 65 years at the time of RFA, the 10‐year OS increased to 74%.

Multiple studies have reported predictors of early recurrence of patients with HCC undergoing RFA [26–28]. Our study is different from previous studies because we used a two‐stage approach to predict prognosis. At baseline, we used a nomogram to predict who could develop true early recurrence or die within two years after RFA. After two years of follow‐up, we could predict prognosis according to recurrence status in the first two years after RFA.

The strength of our study is that we were able to confirm the vital status of every patient. In addition, our study defined true early recurrence as the development of nonlocal recurrence within two years after the initial RFA, whereas previous studies did not differentiate between local recurrence and early recurrence [26–28]. However, our study has several limitations. First, its retrospective design might have led to selection bias. Second, the C‐index of our nomogram was not satisfactory. Future studies using radiomics to predict MVI of HCC [26] may improve the performance of our model. Third, RFA was performed by multiple operators, and there was interoperator variability in technique. Finally, to appropriately exclude local recurrences from the data, we had to either clarify an adequate ablative safety margin on the imaging or clearly categorize the recurrences as local or intrahepatic at distant sites. Because the adequate ablative safety margins were not detailed in our imaging reports, we strictly defined and separated the recurrences into “local recurrence” and “IDR.” We reassigned 34 patients who experienced local recurrence from their “true early recurrence” group (Group 1) into Group 2. However, whereas the current standard of care guidelines for thermal ablation of liver tumors explicitly recommend recording of the ablation zone margin from the margin of the treated tumor [29–31], and the present study’s dataset is retrospective and obtained from a time when tools for confirming the biological rationale of the ablation margin, stereotactic targeting, or image overlay and ablation were not yet available. Future studies should include technical factors (i.e., an adequate ablative safety margin confirmed by fusion image systems) in recurrence weighting during routine clinical practice.

## 5. Conclusion

We developed a nomogram to predict true early recurrence or death within two years in patients with HCC after RFA. After two years of follow‐up, we could forecast the prognosis of patients based on their status of recurrence. Consequently, we were able to dynamically inform patients about their prognosis.

A preprint has previously been published [32].

## Author Contributions

Yi‐Hao Yen: conceptualization, methodology, data curation, writing–original draft, writing–review and editing, visualization, and funding acquisition. Kwong‐Ming Kee, Chao‐Hung Hung, Chien‐Hung Chen, Tsung‐Hui Hu, Jing‐Houng Wang, and Sheng‐Nan Lu: supervision.

## Funding

This study was supported by CORPG8N0271 from the Chang Gung Memorial Hospital‐Kaohsiung Medical Center, Taiwan.

## Disclosure

A preprint has previously been published (https://www.researchsquare.com/article/rs-3938340/v1).

## Ethics Statement

The Institutional Review Board of our institution approved this study (approval number: 202400371B0) and waived the need for informed consent.

## Consent

Please see the Ethics Statement.

## Conflicts of Interest

The authors declare no conflicts of interest.

## Data Availability

The data that support the findings of this study are openly available in Dropbox at https://www.dropbox.com/scl/fi/0ju5n7x6dobfuhirphgzb/raw-data-Risk-group-stratification-for-true-early-recurrence-af.xlsx?rlkey=sqkodkzwlhncfv1j7ismlah1f&st=a18kxco8&dl=0.
